# 

**Published:** 1878-10

**Authors:** 


### CONTENTS.

ORIGINAL.

ART. I.—Rachitis. By Joseph Fowler, M. D., 81
ART. II.—Proceedings Buffalo Medical Association, 95
ART. III.—Dermoid and Ovarian Cysts. By Dr. C. H. Eames, 98

MISCELLANEOUS.

Effects of Medicine in small doses, 101
Quarantine, 109
Edson's New Ear Trumpets, 113
Diphtheria and Sewer Gas, 113

EDITORIAL.

The Yellow Fever Epidemic, 114
Protest from Dr. H. R. Storer, 117
Popular Science Monthly, 117
BOOKS REVIEWED:
Anatomy—Descriptive and Surgical, 118
A New Enterprise, 119
Books and Pamphlets Received 119

